# Molecular Basis of NDT-Mediated Activation of Nucleoside-Based Prodrugs and Application in Suicide Gene Therapy

**DOI:** 10.3390/biom11010120

**Published:** 2021-01-18

**Authors:** Javier Acosta, Elena Pérez, Pedro A. Sánchez-Murcia, Cristina Fillat, Jesús Fernández-Lucas

**Affiliations:** 1 Applied Biotechnology Group, European University of Madrid, c/ Tajo s/n, Villaviciosa de Odón, 28670 Madrid, Spain; jacosta19.ja97@gmail.com (J.A.); elena.perez2@universidadeuropea.es (E.P.); 2 Division of Physiological Chemistry, Otto-Loewi Research Center, Medical University of Graz, Neue Stiftingtalstraße 6/III, A-8010 Graz, Austria; pedro.murcia@medunigraz.at; 3 Institut d’Investigacions Biomèdiques August Pi i Sunyer (IDIBAPS), 08036 Barcelona, Spain; cfillat@clinic.cat; 4 Centro de Investigación Biomédica en Red de Enfermedades Raras (CIBERER), 08036 Barcelona, Spain; 5 Grupo de Investigación en Ciencias Naturales y Exactas, GICNEX, Universidad de la Costa, CUC, Calle 58 # 55-66 Barranquilla, Colombia

**Keywords:** chemotherapy, suicide gene therapy, nucleoside analogues, 2′-deoxyribosyltransferase, structural bioinformatics, molecular dynamics

## Abstract

Herein we report the first proof for the application of type II 2′-deoxyribosyltransferase from *Lactobacillus delbrueckii* (*Ld*NDT) in suicide gene therapy for cancer treatment. To this end, we first confirm the hydrolytic ability of *Ld*NDT over the nucleoside-based prodrugs 2′-deoxy-5-fluorouridine (dFUrd), 2′-deoxy-2-fluoroadenosine (dFAdo), and 2′-deoxy-6-methylpurine riboside (d6MetPRib). Such activity was significantly increased (up to 30-fold) in the presence of an acceptor nucleobase. To shed light on the strong nucleobase dependence for enzymatic activity, different molecular dynamics simulations were carried out. Finally, as a proof of concept, we tested the *Ld*NDT/dFAdo system in human cervical cancer (HeLa) cells. Interestingly, *Ld*NDT/dFAdo showed a pronounced reduction in cellular viability with inhibitory concentrations in the low micromolar range. These results open up future opportunities for the clinical implementation of nucleoside 2′-deoxyribosyltransferases (NDTs) in cancer treatment.

## 1. Introduction

According to the World Health Organization (WHO), cancer was responsible for around 9.6 million deaths in 2018. Despite the great advances in its treatment, it is expected that this number will nearly double in 2040 [1]. One of the first strategies in cancer treatment was the use of nucleoside analogues as anticancer agents. In 2013, up to 13 purine and pyrimidine antimetabolites had been approved as chemotherapeutic agents by the Food and Drug Administration (FDA) [2]. However, the clinical use of nucleoside analogues as therapeutic molecules is often limited due to several drawbacks, such as insufficient drug concentrations in tumors, systemic toxicity, lack of selectivity for tumor cells, and the appearance of drug-resistant tumor cells [3,4]. 

The use of enzyme prodrug therapy (EPT) can overcome these undesired effects. Directed enzyme prodrug therapy (DEPT) involves the selective activation of non-toxic (or with negligible toxicity) prodrug(s) in tumor tissues (or within the tumor microenvironment) by exogenous enzyme(s). In this sense, this selective activation generates high local concentrations of the toxic drug, which kills tumor cells, thereby reducing systemic toxicity. However, getting a reasonable concentration of exogenous enzymes in tumor tissues is the major bottleneck of DEPT therapies. In this context, two different DEPT strategies can be distinguished: (i) the delivery of genes which encode prodrug-activating enzymes into the tumor cells (gene-directed enzyme prodrug therapy, GDEPT), using virus or non-virus carriers [5,6] and (ii) the delivery of prodrug-activating enzymes in the tumor cells by employing different carriers, such as tumor-associated monoclonal antibodies linked to prodrug-activating enzyme (antibody-directed enzyme prodrug therapy, ADEPT) [7], or the solid matrixes (immobilized-directed enzyme prodrug therapy, IDEPT) [8].

The major advantage of GDEPT over ADEPT or IDEPT strategies is the possibility to express the targeted enzyme inside tumor cells. However, low efficiency in the transduction process or in the protein expression, can be a limitation to gene delivery strategies. However, ADEPT and IDEPT strategies are not a good alternative when the presence of a cofactor (only present inside the cells) is needed for the catalytic reaction. The main problem of these approaches is the difficulty of large-sized antibody–enzyme conjugates or some immobilized enzyme derivatives to penetrate cell membranes. 

The use of purine and pyrimidine salvage enzymes in GDEPT therapies (also known as suicide gene therapies) for cancer treatment has been previously reported [9]. Several examples of GDEPT can be found in literature, including the combined use of the bacterial cytosine deaminase (CD) and the prodrug 5-fluorocytosine [9], the enzyme/prodrug system thymidine phosphorylase (TP) and 5-fluorouracil (or 2′-deoxy-5-fluorouridine) [10], or the combination of purine nucleoside phosphorylase from *E. coli* (*Ec*PNP) and the prodrug Fludarabine 6.

**Figure 1:**
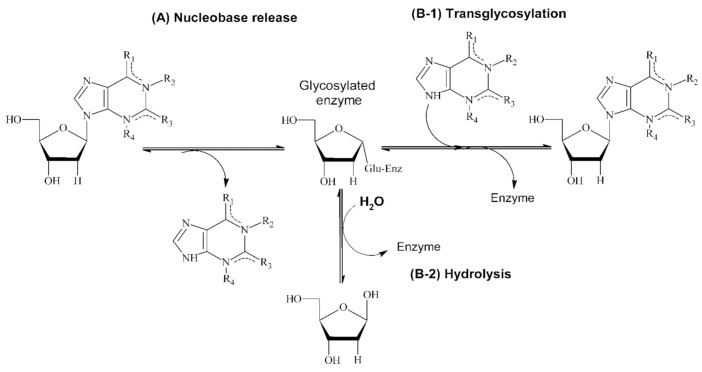
Reaction scheme of NDT-mediated transglycosylation (**A**, **B-1**) and NDT-mediated hydrolysis (**A**, **B-2**).

Nucleoside 2′-deoxyribosyltransferases (NDTs, EC 2.4.2.6), also called *N*-deoxyribosyltransferases or trans-*N*-deoxyribosylases, catalyze the transglycosylation reaction of the 2′-deoxyribose moiety between purine and/or pyrimidine bases (Figure 1) [11,12,13]. Interestingly, in the absence of a second nucleobase the enzyme-glu intermediate is hydrolyzed by a water molecule [14,15] (Figure 1).

NDTs display a strict selectivity for 2′-deoxynucleosides, but noteworthy, they show a high promiscuity in nucleobase recognition which leads to classifying them as type I NDTs (PDT), which catalyze the transglycosylation reaction between purine bases [12,13,16] and type II NDTs (NDT) and which do not discriminate between purines and pyrimidines [17,18,19]. This makes NDTs unique catalysts for the enzymatic synthesis of diverse therapeutic nucleosides [13,20,21,22]. However, with the sole exception of the studies of PDTs from *Borrelia burgdorferi* and *Trypanosoma brucei* as therapeutic targets for Lyme disease [23] and sleeping sickness [24], respectively, no previous studies have looked into the potential of NDTs in biomedicine. 

Herein we report, for the first time, the potential application of NDT from *Lactobacillus delbrueckii* (*Ld*NDT) in suicide gene therapy for cancer treatment. To this end, firstly a functional screening focused on the glycosidase ability of *Ld*NDT with different natural and non-natural nucleosides was performed. Additionally, with the aim of understanding the differences in the specific activity of *Ld*NDT in the presence and absence of nucleobases, molecular dynamics studies were carried out. Finally, the *Ld*NDT/prodrug system was successfully tested as a cell-death inducer in human cervical cancer (HeLa) cells.

## 2. Materials and Methods 

### 2.1. Materials

Cell culture medium reagents were from Difco (St. Louis, MO, USA). Triethyl ammonium acetate buffer was purchased from Sigma-Aldrich (Madrid, Spain). All other reagents and organic solvents were purchased from Symta (Madrid, Spain). Nucleosides and nucleobases used in this work were provided by Carbosynth Ltd. (Compton, UK).

### 2.2. Enzyme Production and Purification

Recombinant His-tagged LdNDT was produced and purified as previously described [22]. The protein concentration was measured by UV_280_ nm spectroscopy assuming the molar extinction coefficient of 34,380 M^−1^ cm^−1^, calculated from the amino-acid sequence.

### 2.3. Substrate Specificity Studies

The standard activity assay to determine NDT-mediated glycosidase activity, in the presence and in the absence of nucleobase acceptor, was adapted from previous literature [12,13,22]. To this effect, 0.3 μg of pure *Ld*NDT was added to a 40 µL solution containing 5 mM 2′-deoxynucleoside (with or without the presence of 5 mM nucleobase) in 50 mM phosphate-buffered saline (PBS) buffer, pH 7.4, for 5–20 min at 37.5 °C and 450 rpm. The enzymatic reaction was quenched by addition of 40 μL of cold methanol in ice-bath and heated for 5 min at 95 °C. After centrifugation at 8500× *g* for 5 min, samples were half-diluted with water and the presence of released nucleobase was determined and quantitatively measured by HPLC (as described below). In such conditions, one activity unit (U) was defined as the amount of enzyme (mg) producing 1 µmol min^−1^ (IU) of released nucleobase under the assay conditions.

### 2.4. Computational Methods

Due to *Ld*NDT having 98% of identity with NDT from *L. leichmannii* (*Ll*NDT), we selected *Ll*NDT as the template for our molecular dynamics (MD) simulations. The Cartesian coordinates of *Ll*NDT in complex with its substrate, 5-methyl-2′-deoxypseudouridine cab be found in the Protein Data Bank server (PDB id. 1F8Y) [25], were used as templates for the complex of *Ll*NDT with 2′-deoxy-5-fluorouridine (dFUrd), namely *Ld*NDT:dFUrd complex, and ribosylated *Ld*NDTcomplex in the presence of 5-flurouracil (5-Fura) at the active site. dFUrd was docked manually into the active site of the enzyme by means of structural best-fit superposition onto the former nucleoside. Care was taken to protonate properly the titratable residues at the active site using the server H++ 3.0 [26]. The ground state geometries of the ligand, dFUrd, the ribosylated glutamic acid, Glu(dRib), 5-flurouracil, and the two adenines tautomers (Ade7 and Ade9), were first optimized in the gas phase and their electrostatic potentials were computed at the standard level of theory (HF/6-31G**//HF/3-21G) and fitted to the atoms as restrained electrostatic potential (RESP) charges using the program antechamber (AmberTools18, URL:ambermd.org). The atoms of the substrates and of the former amino acids were described as AMBER atom types. The leaprcff14SB force field was used in all the MD simulations. The MD simulations were run on GPUs using the pmemd.cuda module of Amber18 in the Single-Precision-Fixed-Precision (SPFP) mode. The *Ll*NDT:dFUrd and ribosylated *Ll*NDT:5-FUra complexes were simulated as protein dimers. In the former complex, dFUrd was placed in each of the active sites; in the latter complex, Glu(dRib) was placed in position 98 and 5-FUra at the active site. Both total complexes were embedded in a truncated octahedral box of *ca*. 14,000 transferable intermolecular potential 3P (TIP3P) water molecules [27] that extended 12 Å away from any solute atom and nine Na^+^ ions were added to ensure charge neutrality. The system was relaxed by energy minimization in three consecutive steps (3 × 5000 cycles), in which after the first 1000 cycles the minimization method was switched from steepest descendent to conjugate gradient. The resulting system was heated from 100 to 300 K during 200 ps with a time step of 0.2 fs and with the position of all the solute atoms restrained with a harmonic potential of 20 kcal mol^−1^ Å^−2^. The Berendsen thermostat [28] was employed for temperature regulation and the simulation was run with a fixed volume (NVT ensemble). The harmonic restraints were gradually reduced in four steps from 40 to 5 kcal mol^−1^ Å^−2^. Then, the density of the system was equilibrated for 20 ps using a time step of 0.2 fs by fixing the pressure, using the Berendsen thermostat with isotropic pressure scaling (NPT ensemble), and allowing the volume of the box to change. Each of the systems were simulated in at least three 100-ns independent simulations (3 × 100 ns) at 300 K with a time step of 2 fs. One of the simulations of the ribosylated system was extended up to 500 ns. A harmonic restraint of 5 kcal mol^−1^ Å^−2^ was imposed on the alpha carbon atoms of the protein in order to maintain the secondary structure. The cutoff distance for the nonbonded interactions was 10 Å and the periodic boundary conditions were used. Electrostatic interactions were treated by using the smooth particle mesh Ewald (PME) method [29] with a grid spacing of 1 Å. The SHAKE algorithm was applied to all bonds involving hydrogen atoms.

### 2.5. Cell Lines and Cell Culture

Human cervical cancer (HeLa) cells were obtained from the American Type Culture Collection, ATCC (Manasas, Virginia, United States), and maintained in Dulbecco’s Modified Eagle Medium (DMEM)+GlutaMax^TM^ supplemented with 10% heat-inactivated fetal bovine serum, penicillin (50 U/mL), and streptomycin (50 µg/mL) in a humidified incubator HERAcell CO_2_ (Thermo Fisher Scientific, Spain) at 37 °C and 5% CO_2_ atmosphere Briefly, cells were grown to confluence as monolayers, trypsinized (0.05% trypsin/0.53 mM EDTA), and plated.

### 2.6. Chemical Transfections

The encoding *ndt* gene, which codifies 2′-deoxyribosyltransferase type II from *L. delbrueckii subsp. lactis* DSM 20072 (NCBI Reference Sequence: WP_002877839.1) was ordered and purchased from Genscript (Piscataway, NJ, USA). The coding sequence was subcloned as *Bam*HI-*Eco*RI fragment into the mammalian expression vector pcDNA3.1+ N-HA, leading to recombinant vector *pLd*NDT_._

As a preliminary study, a first approximation in cell cultures was performed to evaluate the effectiveness of this strategy. For this purpose, a chemical transfection based on the calcium phosphate precipitation method was selected, as it is considered one of the most widely used because it is inexpensive, simple, and suitable for a range of different cell type [30,31]. Thus, cell transfections were performed by CalPhos Mammalian Transfection Kit (Clontech Laboratories, Inc. A Takara Bio Company) in 24-well plates (1 × 10^5^ cells/well) and viability was measured by a MTT colorimetric assay [32]. HeLa cells were transfected with 3µg of *pLd*NDT and 48h after transfection, different concentrations of non-toxic prodrug 2′-deoxy-2-fluoroadenosine (dFAdo) or their respective toxic metabolite (2-fluoroadenine, 2-FAde) were added to evaluate the cytotoxic effect. The absence of mycoplasma and other contaminants was routinely checked in cell cultures.

### 2.7. Analytical Methods

Nucleoside production was analyzed quantitatively with an ACE 5-μm C18-PFP 250 mm × 46 mm column (Advanced Chromatography Technologies) pre-equilibrated in 100% trimethyl ammonium acetate. Elution was carried out by: i) a lineal gradient (0–12 min) from 0.1 M trimethylammonium acetate to 90/10 (*v/v*) 0.1 M trimethylammonium acetate/acetonitrile and ii) an isocratic mobile phase (12–30 min) 90/10 (*v/v*) 0.1 M trimethylammonium acetate/acetonitrile. The flow rate was fixed at 0.7 ml/min (150-bar pressure) and the UV detector was set at 254, 260, 240, and 230 nm. Retention times for the reference natural and non-natural bases (hereafter abbreviated according to the recommendations of the IUPAC-IUB Commission on Biochemical Nomenclature) were as follows: adenine (Ade), 13.3 min; 2-fluoroadenine (2-FAde), 14.7 min; uracil (Ura), 6.8 min; 5-fluorouracil (5-FUra), 7.5 min; and 6-methylpurine (6-MetPur), 15.2 min. Retention times for the nucleosides (hereafter abbreviated according to the recommendations of the IUPAC-IUB Commission on Biochemical Nomenclature) were as follows: 2′-deoxyadenosine (dAdo), 17.2 min; 2′-deoxy-2-fluoroadenosine (2-FdAdo), 19.2 min; 2′-deoxyuridine (dUrd), 10.9; 2′-deoxy-5-fluorouridine (dFUrd), 12.1 min; and 6-methylpurine-2′-deoxyribose (d6MetPRib), 18.9 min. To confirm the reaction products, commercial nucleoside analogues were used as HPLC standards.

## 3. Results and Discussion

### 3.1. Characterization of Glycosidase Activity of LdNDT

**Figure 2:**
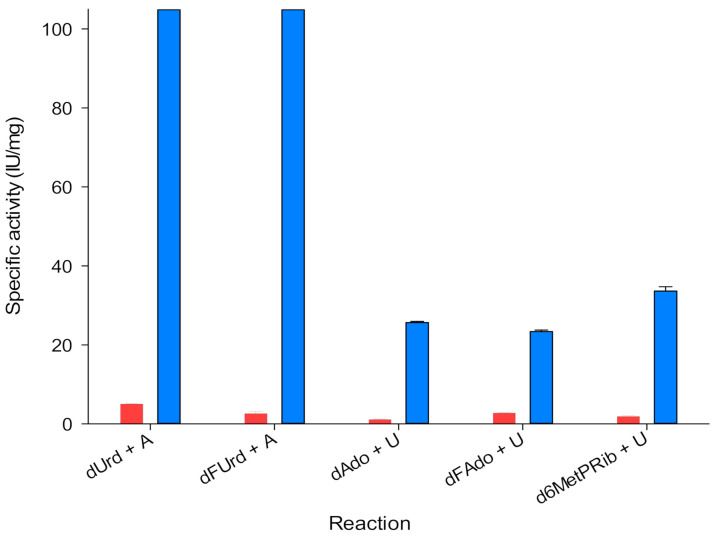
Schematic representation of type II 2′-deoxyribosyltransferase from *Lactobacillus delbrueckii* (*Ld*NDT) glycosidase activity on 2′-deoxynucleosides in the absence (red columns) and presence of nucleobase acceptors (blue columns).

As a starting point, we characterized the C-N glycosidase activity (nucleobase release) of *Ld*NDT on different natural nucleoside (2′-deoxyuridine, dUrd and 2′-deoxyadenosine, dAdo) and several nucleoside-based prodrugs (2′-deoxy-5-fluorouridine, dFUrd; 2′-deoxy-2-fluoroadenosine, dFAdo; and 2′-deoxy-6-methylpurine riboside, d6MetPRib) (Figure 2).

On the one hand, dFUrd, commonly named floxuridine, is a well-known inhibitor of thymidylate synthesis, but is also rapidly converted to the cytotoxic pyrimidine antimetabolite 5-fluorouracil (5-FUra) in mammal cells by thymidine phosphorylase (TyNP) [2,4]. On the other hand, dFAdo and d6MetPRib are potential precursors of the cytotoxic purine antimetabolites 2-fluoroadenine (2-FAde) and 6-methylpurine (6-MetP) [4,33]. Since dFAdo and d6MetPRib cannot be cleaved in mammal cells (human purine nucleoside phosphorylases do not act on 6-amino or 6-methyl purine nucleosides) [33], *Ld*NDT expressing cells could cleave these prodrugs, generating corresponding cytotoxic purine bases, which can also readily diffuse across cell membranes, leading to a bystander activity in surrounding cells (even with low gene expression levels).

Given that, we measured the nucleobase release to the media in the NDT-mediated glycosylation from 2′-deoxynucleoside and base as starting substrates (Figure 2). As a trend, *Ld*NDT shows better activities with pyrimidines (dUrd and dFUrd) than with purines (dAdo, dFAdo, and d6MetPRib). These results agree with those previously reported by Acosta and co-workers [22].

Additionally, we removed the acceptor nucleobase of the media and we measured the release of the nucleobase (Figure 2, red bars). We observed that in these cases the catalytic activity dropped by up to 90%. This means, that nucleobase release is hampered if the acceptor nucleobase is not present, suggesting the presence of a ternary complex—*Ld*NDT + donor (nucleoside) + acceptor (nucleobase).

### 3.2. Molecular Dynamics: Understanding Nucleobase Release

**Figure 3:**
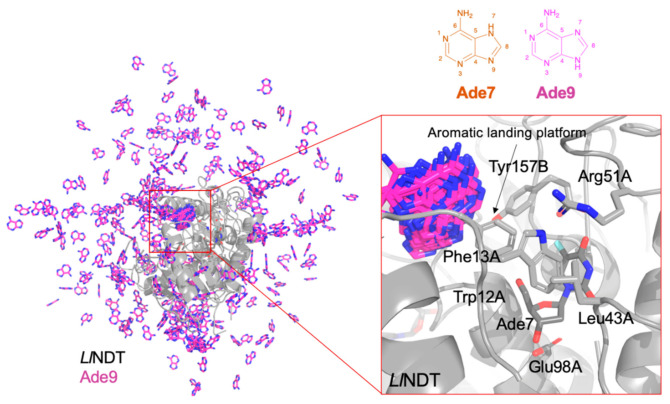
NDT from *L. leichmannii* (*Ll*NDT): 2′-deoxy-5-fluorouridine (dFUrd) simulation in the presence of the two main tautomers in solution of adenine (Ade7 and Ade9) in a box of water molecules. As an example, the different solutions for AD9 are shown. The major cluster of bound nucleobases is highlighted in the zoom-in of the active site—AD9 binds at the aromatic landing platform defined by the side chains of Phe13A and Tyr157B.

With the aim of shedding a light on the experimental data, we ran molecular dynamics (MD) simulation studies to understand how the entering nucleobase could interact with the *Ld*NDT:nucleoside complex. To this end, we selected as system of study the complex type II 2′-deoxyribosyltransferase from *Lactobacillus leichmannii* (*Ll*NDT) with dFUrd (nucleoside donor) and adenine (nucleobase acceptor). *Ll*NDT has a 98% of sequence identity with *Ld*NDT and its structure is available in the Protein Data Bank server (PDB id. 1F8Y) [25,34]. Since the two major tautomers of adenine in solution, Ade7 and Ade9, are protonated on N-7 and N-9 (Figure 3), respectively, after solvation with water molecules, several adenines in the two protonation states were added to the former system, and three independent MD simulations of 300 ns were computed.

The residues that participate in the catalytic reaction mechanism of these kinds of enzymes were identified some time ago [11,14,35]. In addition, we recently proposed a complete reaction mechanism for the two half-reactions of *Ll*NDT with dIno as substrate [14]. To test that model, we cross-validated the computed energy barriers with experimental data of different enzyme variants.

Nevertheless, there are still several unknowns for the catalytic reaction mechanism of the enzyme, such as how the acceptor nucleobase enters to the active site or, in line with results shown in Figure 2, why the specific activity improves when the acceptor nucleobase is present in the reaction mixture from the beginning.

In Figure 3 a representation of the simulated *Ll*NDT:dFUrd complex in the presence of Ade9 in the solution is depicted. We observed that the entering nucleobase Ade9 (and Ade7 too, data not shown) visits the ‘aromatic landing platform’ close to the active site and that is defined by Tyr157B and Phe13A. No other significant pathways are explored by the entering nucleobase. This is due to the action of the side chains of Trp12A, Leu43A, and Arg51A, which protect the active site and exclude the entrance of the acceptor nucleobase.

**Figure 4:**
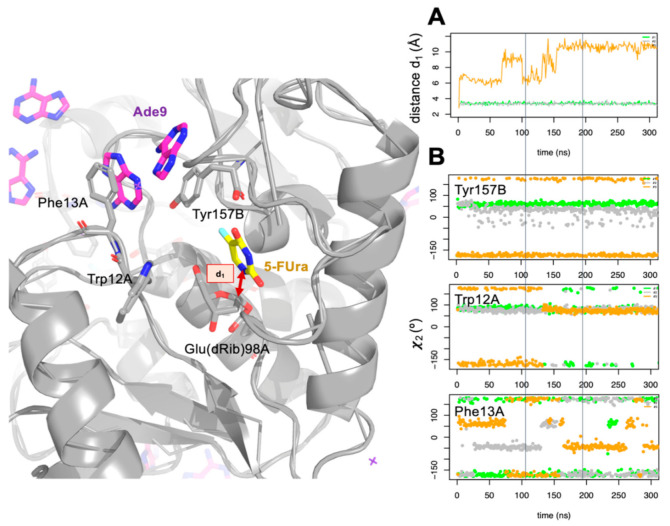
(**A**) Evolution of the distance d_1_ (Å) between N4 of 5-flurouracil (5-FUra) and C1′ of Glu(dRib)98 along the three independent molecular dynamics (MD) simulations (points green, gray, and orange). Distance d_1_ is highlighted on the image on the left with a two-head arrow. Protein is shown as gray cartoons, some relevant residues, and adenosine residues as sticks. (**B**) Dihedral angle χ (°) evolution of the residues Tyr157B, Trp12A, and Phr13A along the three independent MD simulations.

To assess how the presence of the entering nucleobase affects the NDT-mediated glycosylation process, we simulated the *Ll*NDT:dFUrd complex once the enzyme was glycosylated and the 5-FUra was ready to leave in the presence of the entering nucleobase. As before, we ran three independent MD simulations in the presence of both Ade7 and Ade9 (see computational methods). In Figure 4 we monitored the distance between the two atoms that formed part of the glycosidic bond in dFUrd (N4 and C1′) along the three MD simulations. In one of them, we observed how 5-FUra was leaving the active site (orange line, Figure 4A). Visual inspection of this simulation revealed to us that 5-FUra moves to the aromatic landing platform where the adenine residues were previously located (according to previous simulations). Leaving of the nucleobase would share the same pathway as the entering nucleobase. So, this process has to be interconnected. It must be stressed that no water molecule was observed to occupy the site of 5-FUra. The apolar character given by some residues at the active site of *Ll*NDT (e.g., Met125A) prevents the entering of water molecules.

Looking at conformational changes that happened when 5-FUra leaves the active site, we found that Tyr157B, Trp12A, and Phe13A populated different conformer clusters during this process (Figure 4B). To show this, we measured the dihedral angle χ defined as the rotation around the Cß–Cγ bond of the amino acids and that represented the relative orientation of their side chains. After ca. 80 ns of simulation, 5-FUra moved over the 5′-OH of the glycosylated enzyme (Figure 4A). 5-FUra stayed dancing around and after ca. 150 ns it got stuck with Phe13A and Tyr157B until the end of the simulation. Sadly, after 0.5 µs of MD simulation we could not yet see 5-FUra moving to the solvent. Phe13A flipped-in/-out along the trajectory. When pointing to the active site (flipped-in, χ > 100° in absolute value), Phe13A defined the ground of the landing aromatic platform as shown before. When pointing out (flipped-out, χ < 100° in absolute value) there was space for the nucleobase to leave the active site. Altogether, our results point out that the presence of the entering base at the beginning of the catalytic reaction may affect the conformational space visited by Trp12A, Phe13A, and Tyr157B and that the entering/leaving of the nucleobases should be encompassed processes.

### 3.3. Application of NDT/Prodrug Systems in Suicide Gene Therapy

The early cytotoxic effect of the generated compound 2-FAde was demonstrated in previous studies [33,36], where results revealed a great effect of this compound in the inhibition of DNA synthesis, followed by RNA and protein synthesis interruption, respectively. Unfortunately, 2-FAde displays a remarkable cytotoxicity in both cancer and non-cancer cells, even at short concentrations [6], which precludes its clinical use as a chemotherapeutic agent. Due to the fact that human purine nucleoside phosphorylase (*Hs*PNP) does not act on 6-aminopurine nucleosides, the use of GDEPT based on the *Ld*NDT/dFAdo system would allow the selective activation of dFAdo in tumor cells only [4,6,33], and this cytotoxicity should be restricted only to the tumor microenvironment. Considering all the above mentioned features, we hypothesized about the use of NDT/prodrug systems in suicide gene therapies for cancer treatment.

To this aim, HeLa cells were chemically transfected with the pcDNA3.1 + N-HA*_Ld_*_NDT_ (p*Ld*NDT) plasmid vector expressing *Ld*NDT under the control of the cytomegalovirus promoter and treated with dFAdo (prodrug). Since the toxic metabolite, 2-Fade, is generated upon catalysis of dFAdo, cells were also treated with 2-FAde for comparative analysis.

**Figure 5:**
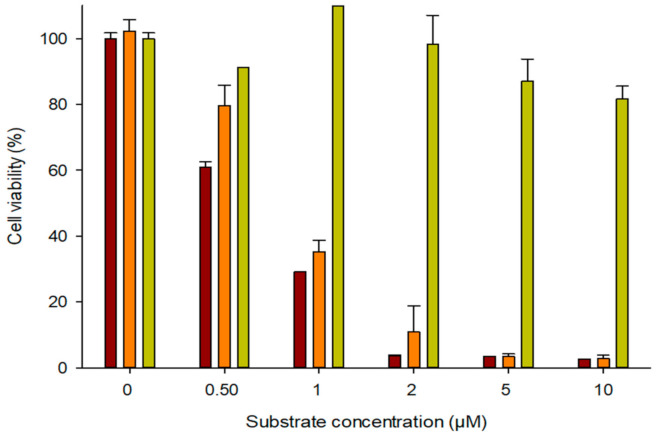
Cytotoxic effect of 2-fluoroadenine (2-Fade) and 2′-deoxy-2-fluoroadenosine (dFAdo) in wild-type and *Ld*NDT transfected HeLa cells.(■) Wild-type HeLa cells + 2-Fade, (■) *Ld*NDT transfected HeLa cells + dFAdo, and (■) wild-type HeLa cells + dFAdo.

According to the experimental results, HeLa cell treatment with the toxic compound 2-FAde for 24 h resulted in a concentration-dependent decline in viability (Figure 5). At 0.5 µM and 10 µM 2-FAde exposure cell viability was 61.04% and 2.64%, respectively. In contrast, treatment with 0.5, 1, or 2.5 µM of dFAdo did not affect cell survival and concentrations of 5 and 10 µM had a minimal effect on cell viability (87.01% and 81.64%, respectively). The IC_50_ of 2-FAde and dFAdo in wild-type HeLa cells was 0.65 µM and 70 µM (data not shown), respectively. In this sense, whereas experimental results revealed that dFAdo did show negligible effect on non-transfected cells (wild-type HeLa cells), 2-FAde showed a remarkable cytotoxicity, even at short concentrations (as expected) [6]. These results illustrate the unharmful effect of the dFAdo prodrug in this in vitro model.

Interestingly, transfection of HeLa cells with p*Ld*NDT followed by dFAdo prodrug treatment revealed significant cytotoxicity at 1 µM dFAdo. Of note, at this concentration similar cytotoxicity was achieved with p*Ld*NDT/dFAdo and 2-FAde, highlighting the efficiency of the enzymatic activity in transforming dFAdo into 2-FAde (Figure 5). These results indicate that the pro-drug dFAdo diffuses across cell membranes of tumor cells and is effectively metabolized to the diffusible cellular toxic compound 2-FAde within 24 h in cells that express *Ld*NDT.

**Figure 6:**
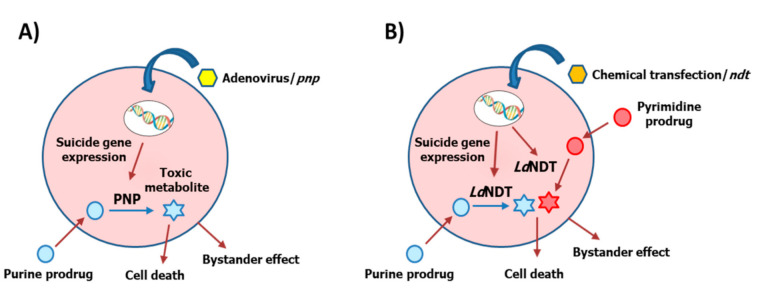
Different protein-/nucleo-based-prodrug gene-directed enzyme prodrug therapy (GDEPT) systems. (**A**) Enzymatic hydrolysis of purine prodrugs by PNP and (**B**) enzymatic hydrolysis of purine and/or pyrimidine prodrugs by *Ld*NDT.

Considering that the transfection efficiency of the HeLa cells was probably not too high, which limited *Ld*NDT expression to a small percentage of the cells in the culture, we hypothesize that a high bystander effect might occur. This will be in accordance with results from the *Ec*PNP/prodrug system [6,33,36,37,38,39]. Similarly to what these authors observed, fludarabine and 6-methylpurine might also be good prodrug compounds for the *Ld*NDT/prodrug GDEPT (Figure 6A). Additionally, the promiscuity of *Ld*NDT (able to act on purine and pyrimidine nucleosides) would expand to (i) pyrimidine nucleoside prodrugs (5-fluorouracil nucleosides) (Figure 6B) and (ii) purine/pyrimidine prodrug cocktail (Figure 6B). This high versatility would increase the possibility of attacking tumor cells. Moreover, according to our results *Ld*NDT/dFAdo could be suggested as an efficient alternative to the PNP/prodrug system, which would offer a broad range of chemotherapeutic treatments.

## 4. Conclusions

Taking into account all these features, intracellular activation of prodrugs by *Ld*NDT provides a selective death of tumor cells expressing the transfected *ndt_Ld_* gene. Our results display the great potential of a GDEPT system based on *Ld*NDT/dFAdo for the treatment of several cancers (e.g., solid tumors). Recently, the FDA has granted orphan drug status to Gedeptin^TM^ (adenoviral vector-expressing *E. coli* PNP gene), that works efficiently with a broad range of nucleoside prodrugs generating active metabolites with high anti-tumor activity for treatment of anatomically accessible oral and pharyngeal cancers. In this sense, it could pave the way for future clinical implementation of NDT/suicide gene therapy. However, several aspects, such as (i) developing a more appropriate transfection system (e.g., viral gene delivery systems), (ii) getting optimal gene expression, for instance by means of tumor-specific promoters, (iii) achieving high-levels of cytotoxic metabolite into transfected cells, and mainly, (iv) evaluating their potential preclinical activity in mouse models, require further investigation and clarification prior to thinking of clinical implementation.
